# The impact of acquired brain damage in terms of epidemiology, economics and loss in quality of life

**DOI:** 10.1186/1471-2377-11-46

**Published:** 2011-04-18

**Authors:** Javier Mar, Arantzazu Arrospide, José María Begiristain, Isabel Larrañaga, Elena Elosegui, Juan Oliva-Moreno

**Affiliations:** 1 Clinical Management Service, Alto Deba Hospital, Mondragon, Spain; 2 Health Research Unit (West Gipuzkoa), Alto Deba Hospital, Mondragon, Spain; 3 Gipuzkoa Regional Office for Health, Government of the Basque Country, Donostia-San Sebastian, Spain; 4 Toledo Faculty of Legal and Social Sciences, University of Castilla la Mancha, Toledo, Spain

## Abstract

**Background:**

Patients with acquired brain damage (ABD) have suffered a brain lesion that interrupts vital development in the physical, psychological and social spheres. Stroke and traumatic brain injury (TBI) are the two main causes. The objectives of this study were to estimate the incidence and prevalence of ABD in the population of the Basque Country and Navarre in 2008, to calculate the associated cost of the care required and finally to assess the loss in health-related quality of life.

**Methods:**

On the one hand, a cross-sectional survey was carried out, in order to estimate the incidence of ABD and its consequences in terms of costs and loss in quality of life from the evolution of a sample of patients diagnosed with stroke and TBI. On the other hand, a discrete event simulation model was built that enabled the prevalence of ABD to be estimated. Finally, a calculation was made of the formal and informal costs of ABD in the population of the Basque Country and Navarre (2,750,000 people).

**Results:**

The cross-sectional study showed that the incidences of ABD caused by stroke and TBI were 61.8 and 12.5 cases per 100,000 per year respectively, while the overall prevalence was 657 cases per 100,000 people. The SF-36 physical and mental component scores were 28.9 and 44.5 respectively. The total economic burden was calculated to be 382.14 million euro per year, distributed between 215.27 and 166.87 of formal and informal burden respectively. The average cost per individual was 21,040 € per year.

**Conclusions:**

The main conclusion of this study is that ABD has a high impact in both epidemiological and economic terms as well as loss in quality of life. The overall prevalence obtained is equivalent to 0.7% of the total population. The substantial economic burden is distributed nearly evenly between formal and informal costs. Specifically, it was found that the physical dimensions of quality of life are the most severely affected. The prevalence-based approach showed adequate to estimate the population impact of ABD and the resources needed to compensate the disability.

## Background

Medicine and society as a whole have been more interested in the causes of the diseases that result in mortality than in their consequences in terms of long term disability [1]. In the case of acute lesions and diseases that affect the brain, there is plenty of work in the literature analysing their causes, including stroke and traumatic brain injury (TBI) [2-6]. However, few studies have focused on the overall population outcome of the sequelae following acquired brain damage (ABD). It is recognised that the permanent sequelae caused by TBI and stroke are one of the main causes of disability [7,8] and that the direct costs of stroke range between 2 and 4% of the health expenditure in industrialised countries [5,6]. However the long-term impact on the family and quality of life of the patient has not been sufficiently well documented. Indeed, the informal costs due to disability represent a largely hidden burden on society [6]. Patients with ABD have suffered a brain lesion that interrupts their vital development in physical, psychological and social spheres [9]. Apart from stroke and TBI, there are other less common causes such as brain tumours, types of meningoencephalitis and the multiple causes of brain anoxia. The classification of the clinical types of patients as a function of a set of functional criteria focussed on care is difficult. The most easily recognisable problems are those involving movement and communication. On the other hand, cognitive and behavioural changes are more difficult to identify and, ultimately, it is these which pose the greatest challenges for social and work reintegration [9]. The natural history of these patients varies depending on the aetiology. What they all have in common is a transition to certain level of disability that typically requires specific social and health care and represents a great loss in quality of life [9].

Society assigns a level of priority to each type of health problem and allocates resources accordingly. Experts use sources of epidemiological information (incidence, prevalence, mortality, loss in quality of life) as well as economic data to define the overall impact [10]. However, experts have suggested that the low level of attention paid to disability-generating health problem is in part due to the fact that information regarding the disease is scattered and not readily accessible [11]. The lack of estimates of the long-term impact of the non-deadly conditions, such as ABD, encourages the implicit belief that there is no problem or that their social impact is smaller than it really is [10].

This study was specifically focussed on the consequences of the disability linked to ABD, given that this is the reason why it is a serious health problem. In relation to this, the objectives of the study were to estimate the incidence and prevalence of ABD in the population of the Basque Country and Navarre in 2008, to calculate the costs required for its care, and finally to quantify the loss in health-related quality of life (HRQL).

## Methods

Two methods were used. First, a cross-sectional survey was used to estimate the incidence of ABD and its consequences in terms of costs and loss in quality of life, from the evolution of a sample composed of patients diagnosed with stroke and TBI. Second, a discrete event simulation model was built to reproduce the natural history of both diseases (stroke and TBI). The models included the incidence of stroke and TBI as well as associated mortality in the general population together with the incidence of ABD obtained in the survey to calculate the prevalence of ABD [12].

### Survey for patients with stroke and TBI

The scope of this study was the geographical area of the Basque Country and Navarre, two regions in the north of Spain, with a total population of 2.75 million, the socio-demographic characteristics being similar in the two areas. Thus, the health system covers the full population basically through public hospitals and health centres, the PIB per person is similar, the economy is mainly dedicated to industry and they share an intense trend to ageing. The incidence of ABD was established according to the degree of permanence of the disability. Hospital records are of limited use, since in Spain the main source of information, the Hospital Minimum Data Set, is focused on identifying the causes of hospital admission and not the consequences. In order to identify the incidence, and the economic and Health-Related Quality of Life (HRQL) impact, a survey was used with patients who had been diagnosed with stroke or TBI in five hospitals in the area. Given the similar social and demographic characteristics and health care requirements, it was assumed that the evolution of patients admitted for stroke and TBI in the participating hospitals could be extrapolated to the whole region of study. Using the codes of the International Classification of Diseases (ICD-9-CM), we defined the cases of stroke as the number of hospital admissions with a main diagnosis code in the range 430-436, except for 433.10 and 435, and TBI for codes 800*, 801*, 803*, 804*, between the 850* and 854*, and 959.01. The number of patients that met those criteria was 5,259 for stroke and 1,696 for TBI in the whole population. The survey was carried out between a year and 18 months after hospital admission. A random sample of 510 patients with stroke and 213 with TBI was selected from the lists of patients with these conditions in 2006. The variables collected by the survey were grouped under the following sections: social and demographic variables (age, sex, work situation, occupation, level of education and family living arrangements), scales to measure levels of disability (the Barthel Index [13] and the Lawton and Brody Instrumental Activities of Daily Living (IADL) Scale [14]), support required for carrying out activities of daily living (in a residential home, day centre, formal home care, informal care) and questionnaires concerning quality of life (SF-36 [15] and EQ-5D [16]). Using the administrative records of hospital admissions, patients or relatives were contacted by telephone to arrange an appointment. The study was approved by the Research Committee. The patients were informed previously about the characteristics of the research and its voluntary nature, and all of them had to give their informed consent to be included in the study. The personnel involved in the fieldwork were specifically trained in completion of the questionnaire. In the cases when the patients could not be contacted or declined to participate, the admission medical report was analysed to assess the presence of ABD.

Diagnosis of ABD was established on the basis of the following criteria: Barthel index score lower than or equal to 95; resignation of employment due to disability as a consequence of stroke or TBI; or loss of two points on the Lawton-Brody scale. In the case of the patients who declined to participate or it was not possible to contact, medical reports were reviewed to identify patients who given their functional state at admission would be likely to still present damage after 18 months. Patients were classified according to their level of autonomy on the basis of the following intervals of the Barthel index: 0-20, the group of maximum disability, 25-60, 65-95 and 100, the group of maximum independence.

### Estimation of the economic impact of the patients with ABD

A bottom-up approach was adopted for the estimation of costs, that is, first the costs associated with a sample population with ABD were assessed to obtain an annual cost per patient [17]. Then, the population costs of ABD were calculated, multiplying the costs per patient by the prevalence data obtained through discrete event simulation. Costs were valued for the year 2008.

The ABD cost was considered to be zero for patients who were independent, with no sequelae from their acute event. For patients who, after their acute event, needed help to carry out activities of daily living, the availability of formal and informal support was assessed. By formal support we mean those services that involve payment, while informal support includes the various different types of non-paid support, to compensate for the disability of patients, provided by relatives and friends. The questionnaire included items which measured the situation of patients in terms of capacity to perform activities of daily living and the services received to compensate for their disability. The assignment of a number of hours per patient to the home support service (formal and informal) relied on the answers to the questionnaire given by the caregivers.

Unit costs were obtained from the care providers in the region and were as follows: day centre (€40.94/day), nursing home, i.e., residential home with a high level of care (€77.57/day), residential care home (€62.05/day), other types of residential homes (€50.96/day), home support services (€18.07/hour) and personal alarm system (€86.28/year). The assessment of the unit costs of informal care was based on the revealed preferences method, considering the cost of substituting for the service [18]. To achieve this, the hours of informal care recorded on the questionnaire were assigned the market value of a similar service. The labour cost was estimated at €11.59 per hour on the basis of Section O of the Spanish Quarterly Survey of Labour Costs which applies to "other social activities and services provided to the community; personal services" [19]. The replacement cost method assumes homogeneity between the quality of the professional service and that delivered by informal care. A maximum of 16 hours per day was set for calculation of the number of hours per month devoted by the close relatives to care of the patient. Finally, unit costs were multiplied by resources used for each individual. This final result provided an estimate of the annual cost per patient with ABD according to type of cost (formal or informal), level of disability and nature of the disorder leading to the condition.

### Impact on health of ABD

The section of the questionnaire intended to assess the impact on health was based, on the one hand, on scales to measure disability (Barthel index and IADL Scale), and on the other hand, on health-related quality of life questionnaires (EQ-5D and SF-36). However, ABD does not explain all the loss of function, given that disability may be due to many other causes. In order to distinguish the fraction attributed to ABD, the population-wide prevalence of disability was obtained from the Basque Country health survey and the results of this survey were adjusted to the age structure of patients with ABD. The Barthel index was used to measure the degree to which patients were able to perform basic activities of daily living, in which it is understood that scores below 96 generally imply disability. Further, the ability of patients to carry out instrumental activities of daily living was estimated using the IADL scale.

The EQ-5D questionnaire is a standardised instrument to measure population valuations of their health status with respect to five dimensions (mobility, self-care, usual activities, pain/discomfort, anxiety/depression) and three levels of severity (no problems, some or moderate problems and extreme problems. Its utility values were calculated on the basis of the results obtained with the Spanish population [16,20]. The SF-36 is a health questionnaire designed to assess patients' the level of health, and corresponding quality of life [15,21]. It consists of 36 questions regarding eight aspects of health: physical functioning, role-physical, bodily pain, general health, vitality, social functioning, role-emotional and mental health. Theses dimensions may also be summarised in terms of two components describing physical and mental health status. The results were compared with those of the general population from the Basque Country Health Survey for 2002 [22]. Given the fact that SF-36 scores of the general population change with age, they were adjusted to the age structure of the sample with ABD. Thus, the differences observed were divided according to their origin: ABD and an age effect.

The magnitude of the observed change between the two groups has been assessed by the age-adjusted effect size. It is calculated by dividing the difference of the means of the values of the ABD sample and the general population, by the standard deviation of the baseline value. Cohen defined a effect size of less than 0.2 as non-significant, of between 0.2 and 0.5 as small, between 0.5 and 0.8 as moderate and with values greater than 0.8 as large [23].

### Estimation of prevalence

Despite its importance, the prevalence of diseases such as stroke or TBI is only known through reports from surveys on small samples [24]. In this study, discrete-event simulation models (DESMs) were used to calculate the prevalence of disability in patients who had previously suffered a stroke or a TBI [25]. The use of this type of models is steadily increasing across a wide variety of fields where it is necessary to make economic assessments thanks, in particular, to their ability to accurately reflect disease progression over time and flexibility to obtain the results in the required form. The main advantage of DES methods is that they can accommodate many entities with different characteristics at the same time. Entities are patients or individuals whose pathway representation is the object of the model. When entities come in the system, they have to be characterized using attributes according to which their future will be determined. This flexibility enables the inclusion of all the different age cohorts contained in the population of interest. As the model runs and the time advances, each individual experiences changes related to its attributes. These changes are recorded, and, in the end, they determine the number of incidental events and the number of entities of specific interest (disability prevalence) across time. The probability of events and the duration of processes are derived from random and independent sampling from theoretical (Gompertz, Dirichlet, Beta) and empirical distributions [25,26]. Table 1 summarizes all the parameters used in both models. Using distributions allows running many times the model (200 replications) to obtain confidence intervals by probabilistic sensitivity analysis [25,26]. The model managed the competitive risks by introducing a function obtained by comparison of both times; this means that the next event to occur was the first in time. The model was built using Arena Software version 10 (Rockwell) [26].

**Table 1:** Traumatic brain injury model parameters and stroke model parameters

Traumatic brain injury model parameters					
Parameter		Source	Distribution	Parameters	
TBI incidence	Entity: TBI	TBI rates (HSU)	Empirical		
Barthel index	Assign Barthel group	TBI rates by Barthel index from our study	Dirichlet	Gamma (1,6)	
				Gamma (1,15)	
				Gamma (1,36)	
				Gamma (1,8)	
Sex	Assign sex	TBI incidence by sex and age (HSU)	Empirical		
Age	Assign age	TBI incidence by sex and age (HSU)	Empirical		
Time until death by other causes with	Assign time	Death rates by sex and age (INE)	Gompertz	Men	α = RR*e^-9.36^
					β = 0.085
				Women	α = RR*e^-12^
					β = 0.112

Stroke model parameters					
**Parameter**		**Source**	**Distribution**	**Parameters**	

Stroke incidence	Entity: Stroke	Stroke rates (HSU)	Empirical		
Barthel index for first ever stroke	Assign Barthel group	Barthel Index distribution from our study	Dirichlet	Gamma (1,32)	
				Gamma (1,44)	
				Gamma (1,105)	
				Gamma (1,16)	
Sex	Assign sex	Stroke incidence by sex and age (HSU)	Empirical		
Age	Assign age	Stroke incidence by sex and age (HSU)	Empirical		
Time until death by other causes with	Assign time	Death rates by sex and age (INE)	Gompertz	Men	α = RR*e^-9.36^
					β = 0.085
				Women	α = RR*e^-12^
					β = 0.112
Time until recurrent stroke	Assign time	Calibration	Gompertz	Men	α = e^-9.0^
					β = 0.085
				Women	α = e^-9.7^
					β = 0.090
Barthel index for recurrent stroke from G0 to	G0	Barthel Index distribution from our study	Dirichlet	Gamma (1,2)	
	Death			Gamma (1,9)	
Barthel index for recurrent stroke from G1 to	G0	Barthel Index distribution from our study	Dirichlet	Gamma (1,5)	
	G1			Gamma (1,7)	
	Death			Gamma (1,18)	
Barthel index for recurrent stroke from G2 to	G0	Barthel Index distribution from our study	Dirichlet	Gamma (1,7)	
	G1			Gamma (1,9)	
	G2			Gamma (1,24)	
	Death			Gamma (1,16)	
Barthel index for recurrent stroke from G3 to	G0	Barthel Index distribution from our study	Dirichlet	Gamma (1,13)	
	G1			Gamma (1,18)	
	G2			Gamma (1,48)	
	G3			Gamma (1,49)	
	Death			Gamma (1,32)	

HSU, Health Statistics Unit in the Spanish Government. ABD, acquired brain damage. INE, Statistics Spanish Institute. RR, relative risk. TBI, Traumatic brain injury. HSU, Health Statistics Unit in the Basque and Navarre Governments. INE, Statistics Spanish Institute. RR, relative risk.

**Figure 1:**
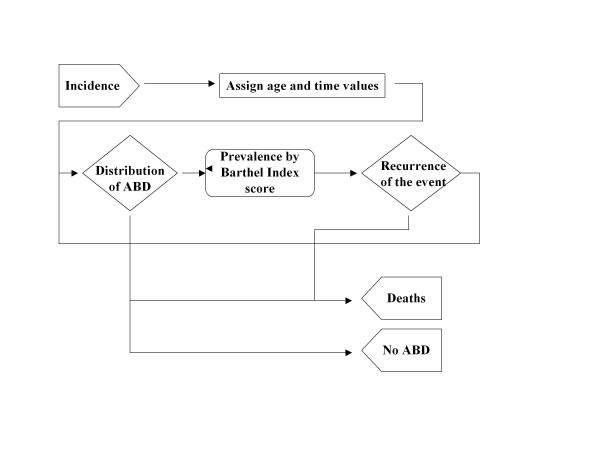
**Conceptual model of ABD starting from the illness causing the condition**. ABD: acquired brain damage.

We used different models for the representation of the natural history of stroke and of TBI, but both held to a similar structure (Figure 1). The model begins by creating new incident cases on the basis of the incidence of stroke and TBI year on year taken from the records of the areas studied. The complete description of the stroke model and its validation has already been published [25]. The incidence of TBI and stroke between 1997 and 2007 was obtained from the Hospital Minimum Data Set for hospitalised patients. These records indicate a hospitalisation rate ranging between 40 and 70 per 100,000 people per year for TBI which is notably lower than those referred to in the literature and it is explained by the decreasing car accidents rate. The annual incidence of stroke varied between 159 and 194 cases per 100,000 people. For this calculation transient ischemic attacks were not taken into account nor were recurrent cases of stroke, which account for 25% of the total figure [7,8]. ABD prevalence is an instant measure defined as the number of individuals in a population which remain disabled after having suffered a stroke or a TBI [27]. Therefore, prevalence changes continuously through time, but we assumed that it remained constant within each year to simplify the presentation of results [25]. The validation of the model was carried out by comparing the outputs with the parameters that were associated with the epidemiology of stroke and TBI in the Basque Country and Navarre.

## Results

From an initial sample of 723 people, a total of 539 individuals were interviewed, of which 361 were diagnosed with stroke and 178 with TBI (Table 2). At the time of the interview, 142 individuals had died. In addition, the interview was not carried out in 31 cases because patients or their relatives declined to participate, and in 11 cases due to changes in address or errors in administrative identification data. In 31 of these 42 cases, the presence of ABD was established from the clinical histories and the admission forms. A total of 282 cases were identified as ABD and this, projected over the total number of patients admitted for stroke and TBI in the populations studied during 2006, gave a rate of ABD of 61.8 per 100,000 people per year due to stroke and 12.5 cases due to TBI.

**Table 2:** Distribution of patients by illness causing the condition and presence of acquired brain damage.

	Stroke		TBI		Total	
	
	Number	Percentage	Number	Percentage	Number	Percentage
ABD	214	42,0	68	31.9	282	39.0
Criteria ABD						
Barthel < 100	173	80.8	55	80.9	228	80.9
Stopped working	1	0.5	2	2.9	3	1.1
Medical Reports	26	12.1	5	7.4	31	11.0
IADL (<4 M, <7 W)	14	6.5	6	8.8	20	7.1

No ABD	173	33.9	115	54	288	39.8
Deceased	116	22.7	26	12.2	142	19.6
Missing information	7	1.4	4	1.9	11	1.5
Total	510	100.0	213	100.0	723	100.0

TBI: traumatic brain damage; ABD: acquired brain injury; IADL: instrumental activities of daily living.

Table 3 shows the characteristics of individuals with ABD by age, sex, work status, Barthel index criteria (for the assignment of ABD) and score obtained in the EQ-5D questionnaire as a function of the Barthel index score. The cases of TBI corresponded to individuals who where younger than cases due to stroke. The scores obtained using the EQ-5D questionnaire fall for smaller values of the Barthel index, and indeed negative values are reached in the group with the highest level of disability.

**Table 3:** Characteristics of the sample with ABD by illness causing the condition.

	Stroke (214 cases)		TBI (68 cases)		Total ABD (282 cases)		No ABD (288 cases)	
	
	Number	Percentage	Number	Percentage	Number	Percentage	Number	Percentage
Age Group								
44 years or younger	3	1.40	7	10.29	10	3.55	64	22.30
From 45 to 54 years	9	4.21	7	10.29	16	5.67	27	9.41
From 55 to 64 years	20	9.35	6	8.82	26	9.22	44	15.33
From 65 to 74 years	44	20.56	11	16.18	55	19.50	75	26.13
From 75 to 84 years	94	43.93	22	32.35	116	41.13	63	21.95
Older than 84 years	44	20.56	15	22.06	59	20.92	14	4.88

Sex								
Men	101	47.20	37	54.40	138	48.90	200	69.44
Women	113	52.80	31	45.60	144	51.10	88	30.56

Employment Status								
Active	0	0.00	2	2.94	2	0.71	39	13.54
Retired	115	53.74	30	44.12	145	51.42	122	42.36
Disabled	13	6.07	11	16.18	24	8.51	8	2.78
Homemaker	34	15.89	8	11.76	42	14.89	14	4.86
Others	10	4.67	5	7.35	15	5.32	42	14.58
NA	42	19.63	12	17.65	54	19.15	63	21.88

Barthel Index								
[0-20]	38	17.80	6	8.80	44	15.60	0	0.00
[25-60]	51	23.80	16	23.50	67	23.80	0	0.00
[65-95]	109	50.90	37	54.40	146	51.80	0	0.00
100	15	7.00	8	11.80	23	8.10	255	88.50
NA	1	0.50	1	1.50	2	0.70	33	11.50

IADL Index								
≥4 M, ≥7 W	38	17.76	17	25.00	55	19.50	246	85.42
<4 M, <7 W	150	70.09	46	67.65	196	69.50	0	0.00
NA	26	12.15	5	7.35	31	10.99	42	14.58

	Mean	SD	Mean	SD	Mean	SD	Mean	SD

EQ-5D								
Barthel's Index [0-20]	-0.28	0.24	-0.21	0.21	-0.27	0.23		
Barthel's Index [25-60]	0.15	0.34	0.36	0.40	0.19	0.36		
Barthel's Index [61-95]	0.51	0.35	0.44	0.34	0.49	0.35		
Barthel's Index 100	0.83	0.20	0.77	0.30	0.81	0.23	0.91	0.17

TBI: traumatic brain injury; ABD: acquired brain damage; M: Men; W: Women.

The annual costs per patient with ABD with respect to the level of disability and the underlying illness are shown in table 4. The average social cost per patient was €21,040/year. This cost of care increases in line with the degree of disability which is characterized by lower Barthel index scores.

**Table 4:** Annual cost per patient with ABD by cost type, disability and illness causing the condition.

	Total annual cost per patient			
Barthel Group	Total ABD	Stroke	TBI	No ABD
[0,20]	44.550	44.270	45.904	
[21,60]	40.813	42.822	34.015	
[61,95]	16.484	17.488	13.725	
[96,100]	3.391	4.268	1.748	413

	Annual formal costs per patient			

Barthel Group	Total ABD	ACVD	TBI	No ABD
[0,20]	35.377	34.274	40.704	
[21,60]	22.992	24.183	18.959	
[61,95]	7.650	8.626	4.965	
[96,100]	1.474	1.394	1.626	398

	Annual informal costs per patient			

Barthel's Group	Total ABD	Stroke	TBI	No ABD
[0,20]	9.173	9.995	5.200	
[21,60]	17.822	18.639	15.056	
[61,95]	8.834	8.861	8.761	
[96,100]	1.917	2.874	122	15

TBI: traumatic brain injury; ABD: acquired brain damage.

Table 5 shows the means for all of the eight SF-36 health dimensions obtained by the patients with ABD. Independent of the illness, it can be seen that the most negative perception of health appears in the dimensions of physical functioning and role-physical (with average scores of 32.4 and 38.8 respectively). The factor with the best perception of health was the role-emotional (score of 74.7), followed by bodily pain (67.2). A comparison of the quality of life of patients with ABD with respect to the general population in the various dimensions of the questionnaire SF-36 is also shown in Table 5, including the crude and the age-adjusted results. The summary "physical" and "mental" scores show that the loss in quality of life in patients with ABD is more marked in the first component, 12.3 versus 6.0. With respect to the physical component, 60.9% of the difference of the quality of life observed between the groups was due to factors linked to ABD. The remaining 39.1% (7.9 points over the observed difference of 20.2) was explained by age-structure differences. The last column of Table 5 indicates that for the physical function and role-physical the effect size was high, while in the dimensions related to mental aspects (role-emotional, mental health), the effect size was only moderate and for pain was minimal.

**Table 5:** SF-36 Questionnaire Dimensions.

	Patients with ABD	General Population (GP)	Observed difference ABD - GP	GP adjusted by age (AGP)	Standardized difference ABD - AGP	Effect of age	Effect size
Physical functioning	32.4	86.9	-54.6	66.4	-34.1	-20.5	1.6
Role physical	38.8	86.3	-47.5	76.0	-37.2	-10.3	1.2
Pain	67.2	77.3	-10.1	69.7	-2.4	-7.7	0.1
Perceived Health	44.1	65.6	-21.6	56.4	-12.4	-9.2	0.6
Vitality	44.3	65.5	-21.1	59.0	-14.6	-6.5	0.7
Social functioning	56.2	88.8	-32.5	82.2	-26.0	-6.6	1.3
Role-emotional	74.7	91.3	-16.7	88.5	-13.9	-2.8	0.5
Mental Health	60.2	72.8	-12.5	69.5	-9.3	-3.2	0.5

Physical component	29.1	49.4	-20.2	41.4	-12.3	-7.9	1.2
Mental component	44.6	50.0	-5.4	50.6	-6.0	0.6	0.6

Comparison of patients with ABD and the general population adjusted by age.

Individuals from the interval [0-15] were not included. ABD: acquired brain damage. GP: General population. AGP: general population adjusted for age; Effect of age: observed difference- standardized difference.

The prevalence according the level of disability by the index of Barthel and by age is shown in table 6. There were an estimated 657 cases per 100,000 people of whom 60% were due to stroke and 40% to TBI. To estimate the economic burden, the cost per patient was multiplied by the prevalence, in each case separating according to Barthel level. In this way, the overall burden is obtained and broken down according to the formal and informal costs depending on the underlying illness (Table 7). The total cost of the burden was estimated at €382.14 million per year, with €215.27 million corresponding to formal burden and €166.87 million to informal burden.

**Table 6:** Prevalence of ABD in the Basque Country and Navarre

STROKE	G0	G1	G2	G3	Sum
From 0 to 15 years	0	0	0	0	0
From 16 to 44 years	0	103	43	240	386
From 45 to 54 years	42	95	326	89	552
From 55 to 64 years	97	217	626	125	1.065
From 65 to 74 years	280	447	1.283	290	2.300
From 75 to 84 years	603	804	2.243	414	4.064
Older than 84 years	371	521	1.418	228	2.538

Sum	1.393	2.187	5.939	1.386	10.905
Upper CI	1.386	2.180	5.924	1.378	10.886
Lower CI	1.400	2.198	5.952	1.391	10.923

TBI	G0	G1	G2	G3	Sum

From 0 to 15 years	0	137	0	94	231
From 16 to 44 years	0	326	308	720	1.354
From 45 to 54 years	0	179	277	292	748
From 55 to 64 years	0	157	350	285	792
From 65 to 74 years	74	185	519	231	1.009
From 75 to 84 years	112	259	895	143	1.409
Older than 84 years	68	375	1.031	486	1.960

Sum	254	1.618	3.380	2.251	7.503
Upper CI	259	1.630	3.395	2.260	7.526
Lower CI	250	1.610	3.367	2.238	7.482

Total	G0	G1	G2	G3	Sum

From 0 to 15 years	0	137	0	94	231
From 16 to 44 years	0	429	351	960	1.740
From 45 to 54 years	42	274	603	381	1.300
From 55 to 64 years	97	374	976	410	1.857
From 65 to 74 years	354	632	1.802	521	3.309
From 75 to 84 years	715	1.063	3.138	557	5.473
Older than 84 years	439	896	2.449	714	4.498

Sum	1.647	3.805	9.319	3.637	18.408
Upper CI	1.658	3.828	9.347	3.652	18.449
Lower CI	1.635	3.790	9.291	3.615	18.368

TBI: traumatic brain injury; ABD: acquired brain damage; M: Men; W: Women. G0: Barthel Group [0, 20]. G1: Barthel Group [21, 60]. G2: Barthel Group [61, 95]. G3: Barthel Group [96,100]. CI: Confidence Interval.

**Table 7:** Total annual costs (formal and informal) of ABD by illness causing the condition (millions €).

Total costs	Stroke		TBI		Total Costs	
	Euros	%	Euros	%	Euros	%
Formal Costs	153.8	58.0	61.47	52.5	215.27	56.3
Informal Costs	111.3	42.0	55.57	47.5	166.87	43.7
Cost Total	265.09	100.0	117.04	100.0	382.14	100.0

ABD: acquired brain damage

## Discussion

This work answers the lack of existing empirical data on ABD in Spain. In particular, it should be emphasised that a substantial number of people experience a loss in their level of independence and quality of life (18,408) which represents 0.7% of the total population. If we see the burden of ABD as an iceberg, the conventional view, based on hospital admission records, only allows us to see the tip that emerges on the surface corresponding to the incidence of stroke and TBI. Our study has quantified the prevalence rate, and it is this that makes it possible to assess the overall extent of the burden of ABD at population level, and its associated costs. In relation to this, the importance given to estimates of incidence and prevalence, and the relationship between them, is being reversed. The traditional epidemiological paradigm has focused on the study of diseases that cause mortality and, since the aim is to control the causes, the relevant measure of disease in question is the incidence as a function of the prevalence of risk factors [27]. However in the new paradigm, the interesting measure is the prevalence of disability as a function of the incidence of diseases such as stroke and TBI [24,28], because the calculation of resources required to take care of disabled patients is dependent on this.

The percentage of deaths is higher for stroke, due not only to the greater severity of this condition, but also to the age distribution for each of the diseases. Specifically, studies carried out in the nineteen-nineties show higher incidence rates of traumatic head injury in younger individuals [2]. However, the combination between a decrease in the number of traffic accidents and the ageing of the population has meant that currently the highest rates of admissions for TBI are in individuals over 65 years old.

The literature reports data on costs of other diseases in Spain. For comparison, the annual cost of AIDS per patients is estimated to be €15,750, the costs associated with Alzheimer €28,198 per year, and with degenerative ataxia, €18,776 per year [29-31]. The mean social cost per patient that we have obtained is €21,040, just a little less than the mean cost per patient with Alzheimer's disease. However it should be taken into account that in the aforementioned studies, health care as well as non-health related costs were included, while in our work only social costs were included. The reason for this focus is that we specifically wanted to highlight the substantial costs due to long term sequelae and to loss in independence with respect to the carrying out activities of daily living in the aftermath of acute events (stroke and TBI).

In the light of these data, it is clear that the care of these patients is a great economic effort shared almost equally between institutions and families. The estimated care costs are equivalent to between 10.4 and 11.7% of the total expenditure on public health in the Basque Country and Navarra in 2008. Costs associated with the pain and suffering caused by the condition were not included, so the estimation given here is conservative. The lack of standards for this type of assessment has led to these costs also being omitted in other studies [31].

In addition, we have opted for using the replacement method for the estimation of the costs associated with informal care, that is, we assessed the services provided by the informal carer taking into account that if they did not provide their services, a professional carer would have had to be hired. Basically, we asked how much the replacing the informal carers would cost. This approach does not take into account the heterogeneity between the quality of professional service and that delivered by informal care or other issues mentioned in the literature such as that of multitasking (the performing of other tasks while taking care of the person with limited independence) [32]. However, given the complexity of assessing the cost of the time of carers, there is no assessment method that is free of theoretical or practical problems [33]. Indeed, the unit cost we have used to assess the hours of informal care (€11.59/hour) can be considered as conservative, when it is considered that putting the replacement method into practice would involve taking on the unit costs of a professional home care provider (€18.07/hour).

As expected, the increase in the level of disability measured using the Barthel index is correlated with an increase in total costs per patients. However, informal costs go down in the group with greater level of disability. This is due to the fact that many more of these individuals are cared for in residential homes, so, the family environment ceases to be pivotal in provision of care. However, it remains the case most patients remain at home, since in only 10% of cases the care is provided in residential homes. This statistic emphasises the reality that the family is still the main provider of care even for patients with low levels of personal independence.

Stroke and TBI represent a great risk of loss of independence since the prevalence of disability in patients who have been admitted for TBI and stroke at some point in their life is 44.0%. As for the degree of disability, the differences in quality of life between individual with ABD and the general populations are very large despite the age adjustment. The only dimension that is not significantly affected is that concerning pain. In the dimensions physical functioning, role-physical, and social function, individuals with ABD report that their capacity to manage their own decisions in the spheres of personal and social independence are greatly affected compared to their contemporaries. The quality of life of the individuals with ABD caused by stroke was found to decrease more severely than cases due to TBI. This difference is particularly striking in the physical dimensions such as physical functioning and role-physical and in social functioning. The summary components of SF-36 fall as low as 28.9 in the physical and 44.5 in the mental component, which in terms of effect size implies a very severe deterioration in the physical quality of life and moderate in the mental component.

At the start of this new century, Spain started with low levels of expenditure on social cover associated with long-term care in comparison to other European countries [34]. In Spain, the family has played a predominant role as the main support network to cover for the needs of disabled people. This phenomenon has been possible thanks to lower proportions of middle aged women being in work, and up to the present day has meant that only subsidiary action has been required from the public sector. That is, only now facing a shortfall or total lack of the family support network, together with the limited financial resources of dependent individuals, the Public Administration has started to fund and provide within the public system the necessary care. However, the establishment of the System for Autonomy and Care for Dependency (SAAD) has changed the legal framework with the recognition of disability care as a right. This law was drafted largely in response to an increase in the demand for formal services due to the fact that the social dynamics (smaller family size, increased number of women working, etc.) make the system of family support that has dominated in Spain unsustainable in the future. This means that a proportion of the estimated extensive family resources (costs of informal care) will have to be substituted in the long term by formal resources funded publically or privately. Thus, a substantial proportion of the estimated costs that have been "invisible" to date will show up in the future, and the budgets and resources must be allocated to meet this social demand.

Despite the fact that cost analysis studies have limitations, the governments of many countries and regions keep encouraging researchers to carry them out. The reason for this is that decision-makers consider that information concerning the financial impact caused by the diseases may be useful input for planning their programmes [35]. It does not substitute but is complementary to epidemiological information on population health problems. The Spanish Ministry of Health and Social Policy has included estimations of the associated cost in the recent policy document entitled "Strategies for Stroke in the National Health System", in order to demonstrate the social significance of this health problem before proposing measures to prevent and care it [36]. This growing level of interest suggests that the ability of cost analysis studies to help us understand the social impact of illness, may mean that they become a useful tool for designing public policies [36,37].

In this way, this information may be taken into account in policymaking, with the first step being make available sufficient epidemiological data to identify the level of importance of the problem under study. Secondly, an assessment needs to be made of the costs of these illnesses and problems they present for the community (using the wide concept of social costs), as a way of considering the loss of social wellbeing caused by them. The third step would be to provide access to information on the technical and human resources that could be applied in policies and interventions addressing these problems. After this, the logical step would be to identify which programmes and cross-sector interventions are efficient, that is those policies that achieve an improvement in life expectancy and in quality of life of the population with using minimum available resources. The following step would be to put into practice said interventions and their subsequent evaluation.

## Conclusions

The main conclusion of this study is that ABD has a high impact in both epidemiological and economic terms as well as loss in quality of life. The overall prevalence obtained is equivalent to 0.7% of the total population. The substantial economic burden is distributed nearly evenly between formal and informal costs. Specifically, it was found that the physical dimensions of quality of life are the most severely affected. The prevalence-based approach proved to be adequate to estimate the population impact of ABD and the resources needed to compensate for the disability.

## Conflicts of interest: none

Study funded by grant BIO05/DCA/001 from the Basque Foundation for Health Innovation & Research supported by the Department of Health of the Government of the Basque Country

## Authors' contributions

JM conceived the study and wrote the manuscript. IL and AA performed the statistical analysis and the systematic review, built the simulation model and helped to draft the manuscript. JMB, EO and JO participated in the design, carried out the survey and helped to draft the manuscript. All authors read and approved the final manuscript.

## Pre-publication history

The pre-publication history for this paper can be accessed here:

http://www.biomedcentral.com/1471-2377/11/46/prepub
